# Optimizing the Use of N-terminal Pro-B-Type Natriuretic Peptide (NT-proBNP) in the Diagnosis of Heart Failure With Preserved Ejection Fraction (HFpEF): A Clinical Pathway Approach to an Underdiagnosed Entity

**DOI:** 10.7759/cureus.99161

**Published:** 2025-12-13

**Authors:** Mukulesh Gupta, Dinesh Kumar, Tuhina Gupta

**Affiliations:** 1 Department of Medicine, Prasad Institute of Medical Sciences, Lucknow, IND; 2 Department of Medicine, Prasad Medical College and Hospital, Lucknow, IND; 3 Obstetrics and Gynaecology, SGT Medical College and Hospital, Gurugram, IND

**Keywords:** biomarkers, clinical algorithms, comorbidities and confounders, heart failure with preserved ejection fraction, nt-probnp

## Abstract

Heart failure (HF) with preserved ejection fraction (HFpEF) constitutes approximately 50% of all global HF cases and remains one of the most underdiagnosed and undertreated cardiovascular conditions. Diagnosis is often delayed because its symptoms overlap with comorbidities in older adults and the inadequate validation of diagnostic tools for HFpEF. N-terminal pro-B-type natriuretic peptide (NT-proBNP) is a recognized biomarker indicative of myocardial wall stress; however, its ideal diagnostic utility in HFpEF is hindered by physiological and comorbid factors, including age, obesity, atrial fibrillation, and renal dysfunction. This review aimed to critically analyze the changing role of NT-proBNP in diagnosing HFpEF, compile recent evidence (2022-2025), and suggest a structured clinical pathway that incorporates adjusted thresholds, clinical scoring systems, and multimodal imaging. By using a step-by-step approach, doctors can improve the diagnostic yield of NT-proBNP, avoid missing or misdiagnosing patients, and speed up the delivery of the right treatment for this complex condition.

## Introduction and background

Heart failure (HF) remains a significant contributor to morbidity, hospitalizations, and healthcare expenditures worldwide. Around half of all HF patients have preserved left ventricular ejection fraction (LVEF ≥50%), a condition referred to as HFpEF. Patients with HFpEF are typically older, more often women, and commonly have comorbidities such as hypertension, diabetes, obesity, and atrial fibrillation (AF) [1]. Despite its prevalence, HFpEF is frequently underdiagnosed or misclassified, as its clinical manifestations, including exertional dyspnea, fatigue, and reduced exercise tolerance, overlap with those of pulmonary and metabolic disorders.

Biomarkers have long played a key role in diagnosing HFwith NT-proBNP measurement combined with echocardiography widely adopted as a noninvasive diagnostic approach. In HFpEF, the utility of NT-proBNP is less clear than in HFrEF (HF with reduced ejection fraction) due to lower absolute elevations and the presence of multiple confounding factors. Recognizing these constraints and enhancing the interpretation of NT-proBNP is crucial for improving diagnostic accuracy in HFpEF. This review examines the biology of NT-proBNP, the unique diagnostic challenges in HFpEF, factors that complicate interpretation, existing guidelines and algorithm-driven strategies, and suggests a pragmatic clinical pathway to enhance its application in HFpEF diagnosis.

## Review

Pathophysiology of HFpEF: implications for biomarker release

HFpEF is primarily characterized by abnormal ventricular-vascular coupling, impaired active relaxation, increased myocardial stiffness, and elevated left atrial/filling pressures, even in the presence of preserved systolic ejection. There is also growing awareness of systemic factors (such as inflammation, microvascular dysfunction, and fibrosis) that increase the likelihood of diastolic dysfunction in the myocardium. The ventricular cavity is often not dilated, and wall stress may be lower than in overt systolic heart failure; as a result, the stimulus for natriuretic peptide secretion can be attenuated. Consequently, patients with substantial elevation in filling pressure may still exhibit only minor increases in NT-proBNP compared to those with HFrEF [2].

The focus from ventricular-vascular coupling and myocardial stiffness has broadened to encompass systemic inflammation, endothelial dysfunction, and comorbidities, including obesity, hypertension, and diabetes. Recent research has brought attention to microvascular rarefaction, myocardial fibrosis, and cellular signaling pathways in the development of HFpEF.

Recent insights underscore that this syndrome encompasses not only intrinsic myocardial abnormalities but also systemic and peripheral factors that diminish overall cardiovascular reserve. The pathogenesis involves reduced physiological reserve due to skeletal muscle abnormalities and vascular endothelial dysfunction, leading to exercise intolerance - a characteristic feature of HFpEF. Additionally, the increased filling pressures in the left ventricle cause the left atrium to change shape and stop working properly. This is linked to bad outcomes, such as a higher risk of atrial fibrillation and pulmonary hypertension later.

Cellular alterations, including elevated myocardial collagen levels and aberrant titin phosphorylation, contribute to increased myocardial rigidity. These intricate and varied mechanisms elucidate the rationale behind the diverse cardiac structural patterns observed in HFpEF patients, including normal ventricular geometry, and underscore the necessity for a comprehensive clinical management strategy that targets both cardiac and systemic factors to enhance patient outcomes [3]. This physiology explains why standard cut-offs from HFrEF populations might not be accurate for HFpEF and why biomarker interpretation needs to be careful.

Biology and clinical utility of NT-proBNP

When ventricular myocytes sense increased wall stretch or stress, they release NT-proBNP, the N-terminal fragment of pro-BNP, in equal amounts to active BNP. NT-proBNP has a longer half-life and is more stable in plasma than BNP, which makes it easier to use in clinical diagnosis. Research indicates that NT-proBNP is associated with left-ventricular end-diastolic pressure and filling pressures, serving as a predictor of unfavorable outcomes in heart failure across various phenotypes [4-6].

The strengths of NT-proBNP include its high negative predictive value, which makes it a reliable tool for ruling out heart failure when levels are low. It also has strong prognostic significance, as elevated concentrations are consistently associated with worse clinical outcomes, including increased rates of hospitalization and mortality. Additionally, NT-proBNP is easy to measure, widely available across clinical laboratories, and provides rapid results, making it a practical and efficient biomarker in routine clinical settings.

But NT-proBNP levels are not an independent diagnostic tool; they are influenced by age, kidney function, obesity, atrial rhythm, and body composition, and they vary across HF subtypes. In HFpEF, small increases and comorbidities are major confounding factors.

Diagnostic performance of NT-proBNP in HFpEF

The diagnostic efficacy of NT-proBNP for HFpEF has been examined in various cohorts and meta-analyses. For instance, a meta-analysis revealed that, for diastolic dysfunction (an indicator of HFpEF), the sensitivity of NT-proBNP averaged approximately 69% and the specificity 85% across 10 studies (I² high) [7]. Additional studies indicate that NT-proBNP exhibits reduced sensitivity in HFpEF compared to HFrEF, especially in cases of obesity or early-stage disease [8,9].

In a study assessing the rule-out threshold (<125 pg/mL) for suspected HFpEF, overall sensitivity was approximately 77%, but only about 67% for patients with a BMI ≥35 kg/m², indicating a heightened risk of false negatives in the obese phenotype [8]. This is in line with research that shows NT-proBNP levels are lower in obese people, which is partly because their hormones and metabolism are not working as well, which makes it even less accurate as a diagnostic tool for this group of people.

NT-proBNP is also a very important marker for predicting the future in HFpEF. A systematic review of 10,158 HFpEF patients found that elevated NT-proBNP levels were associated with a hazard ratio (HR) of 1.80 (95% CI 1.38-2.35) for adverse outcomes; however, no consistent diagnostic threshold emerged across studies [4]. Other large cohort studies corroborate that NT-proBNP predicts elevated all-cause mortality in HFpEF; however, the extensive standard deviations and significant comorbidity burden constrain its independent prognostic significance in this population. NT-proBNP quartiles continue to exhibit a robust correlation with mortality, even following multivariate adjustment, thereby affirming its prognostic significance despite diagnostic constraints [10,11].

Contextual considerations

Atrial fibrillation, age, and renal impairment are examples of comorbidities that can change biomarker levels and test performance, which can change the diagnostic and prognostic value of NT-proBNP in HFpEF. Some research suggests that changing the NT-proBNP cutoffs based on BMI or using lower thresholds (like <50 pg/mL) might make the test more sensitive in obese people, but this would also mean more false positives. Also, NT-proBNP is useful for figuring out risk and guiding treatment, but it shouldn't be the only thing you use to make decisions, especially in HFpEF. It should be used along with clinical assessment and other diagnostic tools. So, NT-proBNP remains a useful part of the diagnostic process, but using a single unadjusted cutoff is not enough, especially in HFpEF.

Confounding factors and suggested interpretations

Major variables affect NT-proBNP levels and should be accounted for when interpreting results in suspected HFpEF. Table 1 presents a summary of confounding factors influencing NT-proBNP interpretation.

**Table 1:** Confounding factors affecting NT-proBNP interpretation AF: atrial fibrillation; BMI: body mass index; HF: heart failure; HFpEF: heart failure with preserved ejection fraction; NT-proBNP: N-terminal pro-B-type natriuretic peptide; RV: right ventricular

Factor	Effect on NT-proBNP	Interpretation/adjustment
Age (older)	Elevation with advancing age independent of HF	Interpret higher baseline values; use age-adjusted thresholds [12,13]
AF	Robust NT-proBNP elevation independent of HF	Use higher cut-offs or focus on imaging/hemodynamics in AF [1]
Obesity (BMI ≥30 kg/m²)	Suppressed NT-proBNP (30-50% lower)	Recognize that low values may not exclude HFpEF; consider imaging or lower thresholds [14]
Renal dysfunction	Elevated levels from reduced clearance	Interpret with caution; follow trends and correlate with kidney function [15]
Pulmonary disease/RV strain	May increase NT-proBNP even without left-HF	Evaluate for primary pulmonary disease and RV load
Sex differences	Slight elevation in women; minor impact	Minimal in isolation, but consider in combined interpretation

These modifiers emphasize that NT-proBNP should not be used in isolation but rather as part of a diagnostic strategy encompassing multiple areas.

Diagnostic algorithms that integrate NT-proBNP

HFA-PEFF Algorithm

The European Heart Failure Association (HFA) put forward the HFA-PEFF algorithm, which is a three-domain scoring system (functional, morphological, and biomarkers) for diagnosing HFpEF. A "major" biomarker criterion is NT-proBNP ≥ 220 pg/mL in sinus rhythm or ≥ 660 pg/mL in AF [16]. Patients with low total scores are not likely to have HFpEF. If their scores are in the middle range, more tests (stress echo, invasive hemodynamics) are needed.

H₂FPEF Score

The H₂FPEF score is easier to understand and is based on six clinical factors: BMI > 30, ≥2 antihypertensive meds, AF, pulmonary artery systolic pressure >35 mmHg, age > 60, and E/e’ >9. NT-proBNP is not directly included in the score; however, the combination of H₂FPEF and NT-proBNP enhances discrimination [2].

Comparative Performance

Research comparing HFA-PEFF and H₂FPEF indicates that HFA-PEFF possesses greater specificity, whereas H₂FPEF is more readily implemented in primary care settings. Adding NT-proBNP as the first step in triage and then using one of these scores is a balanced way to do things [13].

Proposed Clinical Pathway to Optimize NT-proBNP Use

We propose a practical, stepwise clinical pathway. This algorithm emphasizes that NT-proBNP is one component within a broader diagnostic framework and should trigger, not replace, further evaluation. Table 2 presents a stepwise NT-proBNP-guided diagnostic pathway.

**Table 2:** Stepwise clinical pathway for optimizing NT-proBNP utilization in HFpEF diagnosis AF: atrial fibrillation; BMI: body mass index; CKD: chronic kidney disease; DM: diabetes mellitus; E/e′: ratio of early mitral inflow velocity to mitral annular early diastolic velocity; HFpEF: heart failure with preserved ejection fraction; HTN: hypertension; LA: left atrium; LV: left ventricle; LVEF: left ventricular ejection fraction; NT-proBNP: N-terminal pro–B-type natriuretic peptide; TR velocity: tricuspid regurgitation velocity

Step	Action	Decision/next step
Initial assessment	Clinical history (dyspnea, fatigue, exercise intolerance), signs (edema), risk factors (HTN, DM, obesity, AF)	Estimate pre-test probability (use H₂FPEF or clinical gestalt)
Baseline NT-proBNP	Obtain NT-proBNP; document BMI, rhythm, renal status	If NT-proBNP is below adjusted rule-out → HF unlikely; monitor or evaluate alternate diagnosis. If elevated above the adjusted threshold → proceed to echo
Echocardiography	Measure LVEF, LV mass, LA volume, E/e′, TR velocity	If findings show structural/functional changes consistent with HFpEF → probable diagnosis
Intermediate/discordant cases	Low NT-proBNP but high suspicion (e.g., obese) OR elevated NT-proBNP but non-specific comorbidities	Obtain a diastolic stress echo or invasive hemodynamics/refer to a specialist
Complex comorbidities	AF, CKD, advanced age, obesity	Use higher NT-proBNP cut-offs, emphasize imaging/hemodynamics, and trend values over time
Follow-up	If diagnosis made, initiate phenotype-targeted therapy; if unclear, repeat NT-proBNP in 3-6 months, repeat imaging	Use serial NT-proBNP decline/increase to guide management and monitoring

NT-proBNP in Specific Clinical Scenarios

Obesity-Associated HFpEF

Obesity affects as many as half of HFpEF patients and is linked to a decrease in NT-proBNP production, with levels approximately 30-50% lower than those in non-obese individuals experiencing similar cardiac stress [14]. In these cases, a "normal" NT-proBNP does not rule out HFpEF; clinicians should depend more on clinical suspicion and imaging, or use lower cut-offs.

Atrial Fibrillation

AF is prevalent in HFpEF and increases NT-proBNP independently, influenced by atrial stretch and remodeling. In patients with atrial fibrillation (AF), conventional NT-proBNP thresholds become less specific, necessitating the adoption of higher cut-off values or alternative diagnostic methods such as echocardiography or hemodynamics [1].

Chronic Kidney Disease (CKD)

In CKD, NT-proBNP may be elevated due to diminished clearance and volume overload, complicating interpretation. Some guidelines suggest higher cut-offs (for example, two to three times the usual) and stress trends over single values [15].

Older Age and Female Sex

Being older raises the baseline NT-proBNP levels are higher in women than in men for the same amount of cardiac stress, even in healthy people. Age-adjusted thresholds might make the test more specific and cut down on false positives, especially in older women [12,13].

Serial Testing and Monitoring

Even though NT-proBNP is often used as a diagnostic snapshot, serial testing is useful in HFpEF. Trends in NT-proBNP are linked to changes in filling pressures, volume status, and the effectiveness of therapy. For instance, a significant decrease in NT-proBNP following intervention indicates enhanced hemodynamic, while rising levels despite stable symptoms may indicate disease progression or comorbid conditions [5,14]. Adding serial NT-proBNP to outpatient monitoring or telemonitoring clinics for HFpEF may help detect decompensation early and support treatment.

Emerging Biomarkers and Multimodal Approaches

Due to the heterogeneous nature of HFpEF, depending on just one biomarker, like NT-proBNP, is not very useful. Recent advancements have underscored the potential of multi-biomarker panels, which integrate indicators of myocardial stress, fibrosis, inflammation, and metabolic dysregulation, to enhance diagnostic precision and risk stratification. Biomarkers like galectin-3, soluble ST2, and growth differentiation factor-15 (GDF-15) have become useful additions that show fibrosis, inflammatory pathways, and cellular stress. These biomarkers work with natriuretic peptides to show different pathophysiological aspects of HFpEF [17,18]. Additionally, circulating microRNAs (miRNAs) linked to cardiac remodeling and inflammation are emerging as innovative diagnostic modalities, presenting opportunities for earlier and more accurate detection [19,20].

In parallel, imaging biomarkers, including left atrial strain assessed via speckle tracking echocardiography, yield significant information regarding left atrial function and ventricular-atrial interaction, correlating with diastolic dysfunction and prognosis in HFpEF patients [21]. Exercise echocardiography, particularly diastolic stress echocardiography, is increasingly recognized for its ability to unmask hemodynamic abnormalities not evident at rest, and when combined with biomarkers, it improves diagnostic specificity for HFpEF [22].

Recent developments in systems biology and machine learning (ML) have enabled the integration of multi-biomarker data with clinical, imaging, and hemodynamic parameters. Machine learning models, such as random forests and neural networks, can find complicated biomarker patterns and phenotypes in HFpEF. This sets it apart from other types of heart failure and lets them predict outcomes more accurately than older methods [21,23]. AI-based models that look at echocardiographic videos and biomarker profiles show promise for helping doctors make decisions. They could help reduce uncertainty in diagnosis and guide personalized therapy [24].

Although these approaches are promising, they remain largely investigational. Validation in large, diverse populations and integration into cost-effective clinical workflows are necessary before routine clinical implementation. The combination of multi-biomarker panels, advanced imaging techniques, and machine learning algorithms represents a frontier for improving early detection, phenotyping, and management of HFpEF, offering hope for better-tailored therapies and improved patient outcomes.

These approaches show promise, but they are still mostly experimental. Before routine clinical use, it needs to be tested in large, diverse groups of people and added to clinical workflows that are cost-effective. The integration of multi-biomarker panels, sophisticated imaging modalities, and machine learning algorithms signifies a pioneering approach to enhancing the early detection, phenotyping, and management of HFpEF, presenting prospects for more personalized therapies and improved patient outcomes.

International guideline perspectives

European Society of Cardiology (ESC, 2021)

The ESC guidelines say that NT-proBNP levels should be at least 125 pg/mL in non-acute settings and at least 300 pg/mL in acute settings to help with the diagnosis of HF. They also stress the importance of echocardiography and using biomarkers in the HFA-PEFF algorithm [25].

AHA/ACC/HFSA (2022)

These guidelines support the use of natriuretic peptides for both diagnosing and predicting HFpEF, stressing the importance of interpreting them in the right context (like obesity or kidney problems) and using structured algorithms [15].

Asian/Regional Consensus (2024)

New regional guidelines suggest NT-proBNP cutoffs tailored to each population, accounting for differences in ethnicity, body composition, and comorbidities. This shows that "one size does not fit all" [12].

These guidelines converge on the principle that NT-proBNP is essential for HF diagnosis but must be integrated into a comprehensive diagnostic framework.

Health-system implementation

From a health-system standpoint, integrating an NT-proBNP-guided pathway into primary care and referral networks can facilitate the diagnostic process for HFpEF. At the point of care, NT-proBNP testing, along with clinical decision support that takes into account BMI, age, and rhythm, as well as automated referral triggers (like for echocardiography), could help make specialist referrals more timely and appropriate [4]. Cost-effectiveness analyses indicate that early NT-proBNP triage diminishes superfluous imaging and prevents delayed diagnosis, particularly in light of the increasing incidence of HFpEF.

Research directions

Key future research priorities include verifying whether adjusted NT-proBNP thresholds are appropriate for overweight, older, and racially or ethnically diverse populations, ensuring diagnostic accuracy across subgroups. Further priorities involve conducting implementation trials to evaluate NT-proBNP-based diagnostic pathways in real-world primary care and hospital settings. Additional work is needed to integrate NT-proBNP with emerging biomarkers and multimodal imaging within machine-learning diagnostic frameworks. Research should also assess whether NT-proBNP trajectories can help guide therapeutic decision-making and treatment titration in HFpEF. Finally, using NT-proBNP for outpatient screening of high-risk but asymptomatic individuals-such as those with hypertension, diabetes, or obesity-may aid in identifying pre-HFpEF earlier and facilitating timely intervention [26,27].

Key practical takeaways

NT-proBNP remains an important diagnostic tool in suspected HFpEF, but its interpretation must account for key patient-specific factors such as age, BMI, cardiac rhythm, and kidney function. Rather than relying on a single cutoff value, clinicians should use thresholds adjusted for these variables and integrate NT-proBNP results with clinical pre-test probability assessments, such as the H₂FPEF score, and structured diagnostic algorithms like the HFA-PEFF pathway. While a low NT-proBNP level can be useful for ruling out HFpEF, this may not hold in obese individuals, where “normal” levels can still mask underlying disease. Conversely, elevated NT-proBNP levels should prompt further evaluation with echocardiography or advanced diagnostics, as high values alone are not definitive. Serial NT-proBNP measurements may offer additional clarity in uncertain cases. Overall, incorporating NT-proBNP into a broader clinical pathway helps minimize diagnostic gaps and improve resource-efficient evaluation.

## Conclusions

While NT-proBNP remains a valuable biomarker for the diagnosis and management of HFpEF, it works best when used intelligently and based on pathways rather than just looking at single cut-offs. By combining NT-proBNP with clinical scoring, imaging, and adjusted thresholds for important comorbidities, doctors can make more accurate diagnoses, cut down on missed diagnoses, and provide better care to this growing and under-recognized group of patients. As treatment options for HFpEF continue to expand, the importance of NT-proBNP-guided, pathway-based approaches for timely and accurate diagnosis will only increase.
